# A new surgery choice of bilateral laminoplasty for symptomatic three or more-level lumbar canal stenosis in patients over 60 years old: a two-year retrospective study

**DOI:** 10.1186/s12891-025-08940-1

**Published:** 2025-07-14

**Authors:** Yan Jiang, Xu Yan, Su Fu, Jigang Lou, Yuanping Teng, Pengfei Li, Lei Dai, Dongfang Sun, Song Liu, Chunlin Zhang

**Affiliations:** Department of orthopaedics, the first affiliated hospital of Zhengzhou University, Zhengzhou, 450052 China

**Keywords:** Multiple levels lumbar Canal stenosis, Bilateral laminoplasty, Posterior lumbar interbody fusion, Patients ≥ 60 years

## Abstract

**Introduction:**

Multi-level lumbar canal stenosis (LCS) in patients over 60 years is common and frequently complicated by significant comorbidities, making surgical decisions complex. While posterior lumbar interbody fusion (PLIF) is often used, bilateral laminoplasty offers a potential motion-preserving alternative. This study compares outcomes of bilateral laminoplasty versus PLIF for multi-level LCS in this elderly population.

**Methods:**

This retrospective study included consecutive patients >60 years with ≥3-level LCS undergoing bilateral laminoplasty or PLIF at our center. Patients were followed for a mean of approximately 24 months. We first detailed the bilateral laminoplasty technique. Then, outcomes were assessed using Visual Analog Scale (VAS) for pain, Japanese Orthopaedic Association (JOA) score for neurological function, and Oswestry Disability Index (ODI) for quality of life. Perioperative parameters, complications, and comorbidities were analyzed.

**Results:**

Fifty-one patients met inclusion criteria and surveyed (26 laminoplasty, 25 PLIF). Laminoplasty demonstrated significantly shorter operative time (1.8±0.4 vs. 3.2±0.7 hours) and reduced blood loss (102±38 vs. 318±204 ml) compared to PLIF. Transfusion rates were lower with laminoplasty (0% vs 52%). Both groups showed significant improvements in VAS, JOA, and ODI scores at final follow-up. Laminoplasty achieved significantly better final ODI scores. Comorbidity rates were high, but no serious complications were found.

**Conclusion:**

Bilateral laminoplasty is a safe and effective surgical option for patients over 60 years with multi-level LCS, offering comparable clinical improvements to PLIF while providing advantages of shorter operative time, significantly reduced blood loss and transfusion requirements. It represents a valuable motion-preserving alternative in this comorbid population.

**Supplementary Information:**

The online version contains supplementary material available at 10.1186/s12891-025-08940-1.

## Implications for clinical practice

▪ Bilateral laminoplasty offers a viable motion-preserving alternative to PLIF for patients (≥60 years) with multi-level lumbar canal stenosis (≥3 levels), particularly those with high comorbidity burdens (e.g., hypertension, diabetes, cardiovascular disease).

▪ Prioritize laminoplasty when minimizing surgical trauma is critical, as it demonstrated significantly shorter operative times, reduced blood loss and lower transfusion rates compared to PLIF.

▪ Laminoplasty achieves comparable clinical improvements to PLIF in pain relief, neurological function and quality of life.

## Introduction

Lumbar spinal stenosis (LCS) is a common radiographic finding on MRI, though only 5.7–22.5% of cases manifest clinically significant symptoms [1–3]. In symptomatic patients, typically aged ≥ 60 years, spinal canal narrowing compresses neural structures, causing low back pain, leg pain, limb numbness, and weakness. Histologically, age-related disc degeneration begins in the sixth decade as the nucleus pulposus transitions to fibrocartilage [4]. Pathogenesis involves multifactorial degeneration, including disc herniation, facet joint hypertrophy, and ossification/thickening of the ligamentum flavum [5]. Critically, multi-level involvement is frequent: approximately 47.2% of symptomatic patients exhibit ≥ 2-level stenosis, with 23.5% presenting ≥ 3-level disease [6]. This pattern is strongly associated with age-related comorbidities like hypertension, diabetes, osteoarthritis and cardiovascular disease [7], complicating treatment decisions.

Surgical intervention for elderly patients (> 60 years) with ≥ 3-level LCS requires careful risk-benefit analysis, weighing symptom severity, comorbidity burden, quality-of-life impact, and procedural invasiveness [5, 8]. While neural decompression remains the primary goal for severe symptoms [9, 10], evidence comparing surgical efficacy to conservative management is mixed [11, 12]. Existing techniques—including laminectomy and laminotomy—aim for minimal invasiveness [10, 13], yet systematic reviews show inconsistent advantages in long-term outcomes [14]. Several randomized trials evaluated the decompression comparing to non-operative treatment for LCS favoring surgery at the 1- and 2-year follow-up [15] but concluding a similar status at a longer period of time [16]. By 10 years, 23% of surgical LCS patients accepted an additional lumbar spine operation. To reason the worse outcomes in surgical patients, the none-biological status of vertebral structures caused by surgery may develop the insufficient decompression, segmental instability, adjacent segment disease, et al. [17].

To address these limitations, we propose bilateral laminoplasty—a motion-preserving technique achieving symmetrical canal enlargement while restoring near-physiological anatomy. Decompression grooves were positioned medially termed as symmetrical adduction lumbar decompression/SALD. This approach offers distinct advantages for elderly comorbid patients, as demonstrated in our study: significantly reduced operative time, minimal blood loss, negligible transfusion needs. Although laminoplasty variants exist for spinal tumors [18–20], applications in LCS remain limited. Japanese groups described nerve root decompression via “open-door” laminoplasty with screw fixation [21, 22]; our technique innovates by performing longitudinal decompression across all stenotic levels followed by bilateral titanium mini-plate fixation.

In this study, we first detail our bilateral laminoplasty protocol for ≥ 3-level LCS; Then compare perioperative and 2-year outcomes (VAS, JOA, ODI) against PLIF in 51 patients aged > 60 years. We aimed to demonstrate its viability as a safe, effective, and less invasive alternative to fusion procedures in high-risk patients.

## Methods

### Participants

Patients over 60 years old with symptomatic multiple levels of LCS hospitalized from Jan 2021 to Jan 2023 who underwent bilateral laminoplasty or posterior lumbar interbody fusion (PLIF) were retrospectively reviewed. This trial was approved by the ethic committee of the first affiliated hospital of zhengzhou university (Ref Number of 2022-KY-0357-002). This study was conducted in accordance with the Declaration of Helsinki. All participants provided written informed consent for surgery and data inclusion. Eligible patients presented with moderate-to-severe neurogenic claudication or radiculopathy refractory to ≥ 3 months of conservative management, supported by MRI-confirmed multi-level LCS. I.

The inclusion criteria were as followed: (1) Age > 60 years at the time of the first MRI, (2) three to five levels of LCS were presented from L1 to S1 spine region, which can explain the associated clinical symptoms, such as neurogenic claudication, a progressive onset of pain and neuromuscular deficits initiated by walking, (3) a fixed and experienced team which was leaded by the corresponding author conducted the operation. The following exclusion criteria were also applied: (1) less than three levels of LCS on MRI detection (2) Continuous and significant segment instability confirmed by X-ray examination, (3) far-lateral lumbar disc herniation causing the stenosis of intervertebral foramen, (4) Association with fracture, tumor, infection, congenital spine abnormalities or other conditions affecting lumbar spine and other conditions as mental disorders, alcoholism and drug abuse, (5) Incomplete medical document imaging data or refusal to re-visit our hospital for evaluation.

### Surgical operation

**Fig. 1:**
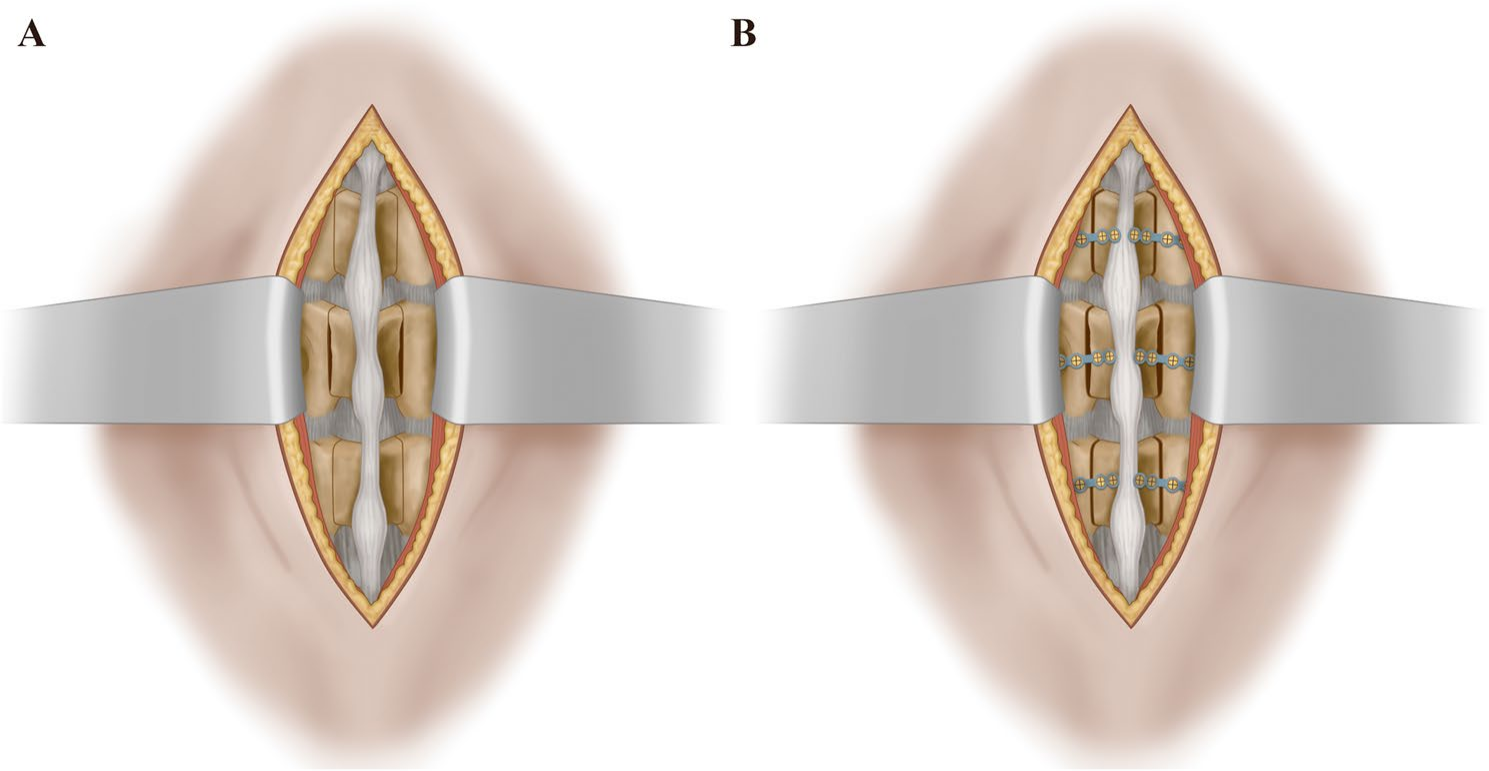
Schematic illustration of bilateral laminoplasty. **A** Paraspinal muscles are dissected and retracted to expose the spinous process and lamina. Decompression grooves are then cut while preserving the supra-/interspinous ligaments and facet joints. **B** Fixation of decompression grooves using two mini-plates with screws

The bilateral laminoplasty protocol is illustrated in Fig. 1. This procedure aims to decompress neural structures through the following steps: exposure, bilateral creation of decompression grooves in the lamina, symmetrical spinal canal enlargement, and fixation using two mini-plates.

Under general anesthesia via tracheal intubation, patients were positioned prone. Fluoroscopy continuously monitored bony landmarks and instrumentation throughout the procedure. A standard posterior midline incision (50–70 mm) allowed subperiosteal dissection of paraspinal muscles from spinous processes and laminae, with lateral retraction preserving the supraspinous and interspinous ligaments. The pars interarticularis and lamina were directly visualized.

The operative field extended from the spinous process to the facet joint (without facet joint resection). After preparing bilateral drill sites adjacent to the spinous process, an ultrasonic osteotome with a 3-mm grinding drill bit created bilateral grooves between the spinous process and medial facet joint border. Grooves (2–3 mm width) were positioned directly over the lateral recess. Ligamentum flavum resection then decompressed nerve roots. The spinous process-ligament complex and lamina were fully mobilized and shifted posteriorly by 3–5 mm to asymmetrically enlarge the spinal canal (Fig. 1A), with subsequent dural sac expansion observed.

For three-level LCS (typically L2-3, L3-4, L4-5), three laminae/spinous processes were addressed. Premolded titanium mini-plates were adjusted for optimal alignment. Medial plate fixation was secured to the spinous process base, while lateral fixation anchored to the pars interarticularis using 2.6-mm screws (Fig. 1B).

A drain was placed prior to layered wound closure. Prophylactic antibiotics were administered for 48 h with continuous neurological monitoring. The drain was removed at 24 h postoperatively. Patients were mobilized with an external brace, which was discontinued at 2 months.

For PLIF, patients were similarly positioned. Through a midline incision, subperiosteal exposure exposed laminae and facets. Bilateral total laminectomy at affected levels was performed alongside medial facetectomy and foraminotomy. Pedicle screws were inserted under fluoroscopy, followed by discectomy, endplate preparation, and insertion of cages packed with local autograft. Rods were secured to pedicle screws under compression. After drain placement and closure, patients mobilized with brace assistance at 48–72 h postoperatively, continuing bracing for 3 months.

### Clinical outcomes measurement

Preoperative and postoperative clinical outcomes were assessed using validated instruments at the final follow-up (mean approximately 24 months). Data collection was performed by independent reviewers.

Pain intensity was quantified using the Visual Analog Scale (VAS, 0–10) for low back and limb pain. Neurological function was evaluated with the Japanese Orthopaedic Association (JOA) scoring system (0–29). Quality of life and functional disability were measured using the Oswestry Disability Index (ODI, 0-100%). The magnitude of improvement was calculated for each scale (postoperative minus preoperative score). Additionally, overall recovery status was categorized based on the standardized JOA improvement grading system (excellent, good, fair, poor). ODI scores were also graded preoperatively and postoperatively to assess functional disability severity.

### Statistical analysis

Continuous variables are presented as mean ± standard deviation (SD), while categorical variables are expressed as frequencies and percentages. Intergroup comparisons (laminoplasty vs. PLIF) were conducted using independent samples t-tests for normally distributed continuous variables (age, follow-up duration, operative time, blood loss, and pre-/postoperative VAS, JOA, and ODI scores), chi-square tests for categorical variables (sex distribution, symptom prevalence, comorbidity rates, transfusion requirements, and complication rates). Intragroup comparisons of pre- versus postoperative clinical outcomes (VAS, JOA, ODI) were performed using paired t-tests. A two-sided *p*-value < 0.05 was considered statistically significant for all analyses. Statistical analyses were executed using SPSS 21.0 (IBM, USA).

## Results

### General information

In this retrospective study, we initially surveyed 92 consecutive patients older than 60 years with three or more levels of LCS who underwent bilateral laminoplasty or PLIF at our medical center. Forty-one patients were lost to follow-up due to loss of contact (37 patients, 40.3%), lumbar spine trauma (1 patient, 1.1%), or death from cancer/heart disease (3 patients, 3.3%). Ultimately, only 51 patients (55.4%) meeting the inclusion criteria at a minimum follow-up of approximately 2 years were identified and enrolled. Among them, 26 underwent bilateral laminoplasty while 25 underwent PLIF. All relevant information was collected and analyzed during the study period.

As shown in Table 1 and SI, 26 patients treated with laminoplasty and 25 patients treated with PLIF returned for follow-up at 24.9 ± 3.8 months and 24.6 ± 4.5 months, respectively (*P* = 0.78). Among the 26 patients (Laminoplasty group), 8 were male (30.8%) and 18 were female (69.2%), with a mean age of 67.6 ± 6.6 years (range: 60–85). In the PLIF group, 8 were male (32.0%) and 17 were female (68.0%) with a mean age of 70.4 ± 6.4 years (range: 60–82). No significant differences were found between groups in sex distribution or age (*P* = 0.15 and *P* = 0.56, respectively).

Patients’ symptom duration was 13.5 ± 18.1 months (laminoplasty group) and 16.6 ± 22.8 months (PLIF group). In the laminoplasty group, the main symptoms were low back pain (20/26, 76.9%), limb numbness (10/26, 38.5%), limb pain (10/26, 38.5%), and limb weakness (5/26, 19.2%). In the PLIF group, the main symptoms were low back pain (18/25, 72.0%), limb numbness (9/25, 36.0%), limb pain (22/25, 88.0%), and limb weakness (5/25, 20.0%). These severe or progressing symptoms prompted them to seek treatment at our center.

Given their age (over 60 years), a large proportion of patients exhibited comorbidities: 88.5% (laminoplasty) and 88.0% (PLIF). In the laminoplasty group, hypertension was the most common comorbidity (14/26, 53.8%). Other comorbidities included diabetes or coronary atherosclerosis (both 5/26, 19.2%), osteoporosis, cerebral infarction, femoral head necrosis, cancer, ankylosing spondylitis, and nephropathy. In the PLIF group, hypertension was also the most common comorbidity (12/25, 48.0%). Other comorbidities included diabetes (7/25, 28.0%), coronary atherosclerosis and osteoporosis (both 4/25, 16.0%), osteoarthritis, and ankylosing spondylitis. No statistical differences in comorbidities were found between groups. Postoperatively, all patients were ambulatory after drain removal, within 2 or 3 days.

### Surgery details

In the laminoplasty group, all 26 patients had over 3 levels of lumbar stenosis: 14 had 3 levels (53.8%), 9 had 4 levels (34.6%), and 3 had 5 levels (11.5%). Based on radiographic signs of LCS, laminoplasty was performed at the stenotic levels to enlarge the spinal canal. The most common operative levels were L3, L4, and L5 (53.8%). No serious technical problems occurred during surgery. Profound facet hypertrophy and osteophyte formation were present in 7 patients (26.9%, *corrected from 15.2% as 7/26 = 26.9%*), complicating the procedure. Occasionally during fixation, one or two screws could not be placed, though this was not deemed critical. All screws and plates were correctly positioned and fixed. Manual testing confirmed stability of the spinous process and lamina. At final follow-up, no infections, neurological deficits, wound dehiscence, or implant failure/loosening were noted. In the PLIF group (25 patients), 11 had 3 levels of LCS (44.0%), 13 had 4 levels (52.0%), and 1 had 5 levels (4.0%). The distribution of LCS levels in the PLIF group was similar to the laminoplasty group. Similarly, no serious technical problems occurred during PLIF surgery.

In the laminoplasty group, operative time was 1.8 ± 0.4 h and total blood loss was 102 ± 38 ml, indicating a rapid procedure with minimal blood loss. Operative time for PLIF was significantly longer at 3.2 ± 0.7 h (*P* < 0.001). Blood loss in the PLIF group was 318 ± 204 ml, significantly greater than in the laminoplasty group (*P* < 0.001). No patients required intraoperative transfusion; however, a higher proportion of PLIF patients received postoperative transfusion (13/25, 52.0%). Among these 13 PLIF patients: 5 received 4 units of packed red blood cells (PRBCs) and 400 ml fresh frozen plasma (FFP); 2 received 300 ml FFP alone; 3 received 100 ml FFP alone; and 3 received 4 units PRBCs alone. The need for transfusion was lower in the laminoplasty group compared to the PLIF group.

### Clinical outcomes

At final follow-up, both groups experienced substantial improvements in pain, neurological function, and quality of life. In the laminoplasty group, mean VAS for low back/limb pain improved significantly from 6.2 ± 1.2 to 2.8 ± 1.7 points. Similarly, the JOA score improved from 19.9 ± 4.0 to 24.9 ± 2.5 points. Based on JOA improvement criteria, recovery was excellent/good in 20 patients (76.9%) and fair in 6 patients (23.1%). The ODI score decreased from 34.8 ± 8.9% to 25.4 ± 6.4%. Preoperatively, 16 patients (61.5%) had moderate and 9 (34.6%) had severe dysfunction. At follow-up, no patients had severe dysfunction, while 20 (76.9%) had moderate dysfunction. In the PLIF group, mean VAS for low back/limb pain also improved significantly from 6.5 ± 0.9 to 3.5 ± 1.7 points, and the JOA score improved from 19.6 ± 2.6 to 23.6 ± 3.5 points. ODI scores improved obviously (from 36.0 ± 9.8% to 30.8 ± 11.5%). However, the final ODI score differed significantly between groups, with greater quality-of-life recovery observed in the laminoplasty group compared to the PLIF group. (Implied based on lower final ODI in laminoplasty).

**Table 1:** Demographic and clinical Information for patients

**Variables**	**Patients (Laminoplasty)**	**Patients (PLIF)**	***P*** ** value**
Age (years)	67.6±6.6	70.4±6.4	0.15
Gender (male)	8 (30.8%)	9 (36%)	0.56
Duration of illness (months)	13.5±18.1	16.6±22.8	0.59
Follow-up time (months)	24.9±3.8	24.6±4.5	0.78
Comorbid conditions	23 (88.5%)	22 (88%)	0.99
Hypertension	14(53.8%)	12 (48%)	0.78
Diabetes	5 (19.2%)	7 (28%)	0.52
Coronary atherosclerosis	5 (19.2%)	4 (16%)	0.99
Osteoporosis	2 (7.7%)	4 (16%)	0.67
Cerebral infarction	2 (7.7%)	0 (0%)	0.49
Femoral head necrosis,	2 (7.7%)	0 (0%)	0.49
Cancer	2 (7.7%)	0 (0%)	0.49
Ankylosing spondylitis	2 (7.7%)	1 (4%)	0.99
Nephropathy	1 (3.8%)	0 (0%)	0.99
Osteoarthritis	0 (0%)	3 (12%)	0.11
Total blood loss (ml)	102±38	318±204	<0.001***
Intraoperative transfusion	0 (0%)	13 (52%)	<0.001***
Operation time (h)	1.8±0.4	3.2±0.7	<0.001***
Levels of LCS/operation			
3	14 (53.8%)	11 (44%)	0.35
4	9 (34.6%)	13 (52%)	
5	3 (11.5%)	1 (4%)	
Clinical outcomes			
VAS			
Pre-operative	6.2±1.2	6.5±0.9	0.53
Post-operative	2.8±1.7	3.5±1.7	0.13
JOA score			
Pre-operative	19.9±4.0	19.6±2.6	0.77
Post-operative	24.9±2.5	23.6±3.5	0.96
ODI score (%)			
Pre-operative	34.8±8.9	36±9.8	0.66
Post-operative	25.4±6.4	30.8±11.5	0.04*

### Comorbid conditions

Given the characteristics of old age and multi-level LCS, complications potentially related to comorbidities were likely. In the laminoplasty group (88.5% comorbidities), conditions mainly included hypertension (53.8%), diabetes, and coronary atherosclerosis (19.2% each). Perioperatively, among 14 patients with hypertension, blood pressure remained stable (< 160/100 mmHg) in only 6; 3 had readings reaching 180/110 mmHg. Of 5 diabetic patients, 2 had high blood glucose (> 10 mmol/L). Five patients had coronary atherosclerosis. Despite these comorbidities, no strokes occurred. However, one patient with hypertension, diabetes, and nephropathy was transferred to the ICU on postoperative day 1 due to low SaO2 and recovered after 3 days of supportive care. Three months postoperatively, 2 patients were rehospitalized for management of cerebral infarction progression (1) and lymphoma (1). At the last follow-up, 4 patients (15.4%) reported persistent postoperative pain with altered location and frequency, attributed to comorbid femoral head necrosis (2 cases) and ankylosing spondylitis (2 cases).

In the PLIF group, blood pressure and glucose remained stable during surgery. However, three patients (12.0%) were transferred to the ICU. One 75-year-old female was diagnosed with nosocomial pneumonia; due to low SaO2, elevated CRP and procalcitonin postoperatively, she received intensive care for 3 days before returning to the ward. The other two patients were transferred due to low SaO2; one returned to orthopedics after 2 days, while the other required 7 days of life support.

### Representative cases

**Fig. 2:**
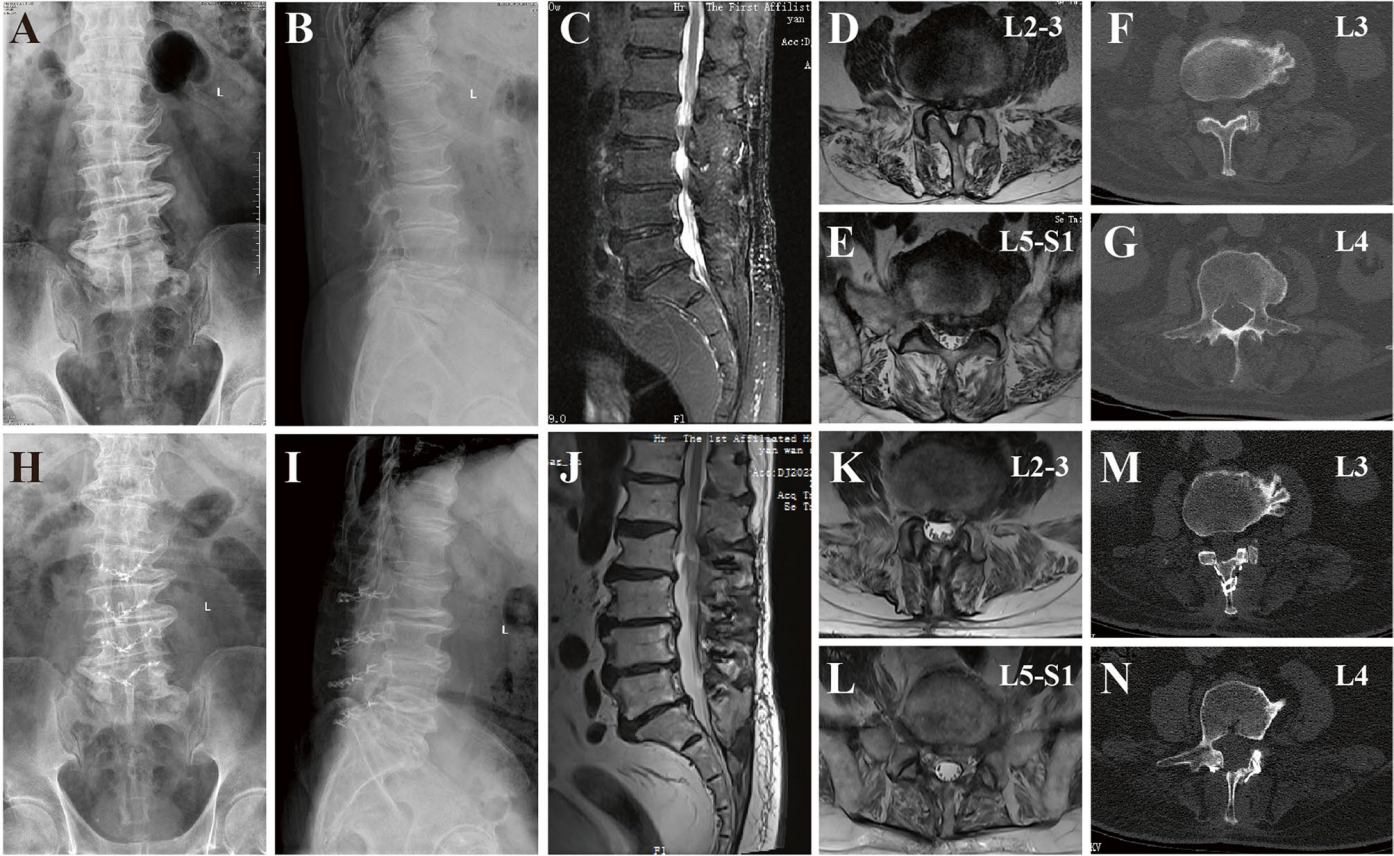
Representative case. A 74-year-old male with 5-level lumbar canal stenosis (LCS) underwent lumbar laminoplasty. Pre-operative imaging (A-G) includes X-rays (**A**, **B**) demonstrating extensive osteophyte formation, an MRI (**C**) showing LCS at five levels with axial views confirming neural compression at L2-3 (**D**) and L5-S1 (**E**), and CT axial views revealing spinal canal narrowing at L3 (**F**) and L4 (**G**). Post-operative imaging at 26 months (H-N) shows X-rays (**H**, **I**) of the laminoplasty fixation, an MRI (**J**) demonstrating significant neural decompression with axial views at L2-3 (**K**) and L5-S1 (**L**) confirming resolution of compression, and CT axial views (**M**, **N**) revealing bony regrowth/reunion within the laminoplasty grooves at L3 and L4 levels

A representative case of bilateral laminoplasty is shown in Fig. 2. A 74-year-old male presented with limb pain and numbness for 3 years. As conservative treatment failed, he underwent laminoplasty. Initial assessment X-rays showed extensive osteophytes (Figs. 2A, B). MRI revealed LCS from L1 to L5 due to disc herniation and ligamentum flavum hypertrophy (Fig. 2C), with neural compression at L2-3 and L5-S1 (Figs. 2D, E). Spinal canal anatomy at L3 and L4 is shown (Figs. 2F, G). At 26-month follow-up, limb pain and numbness were greatly improved. X-rays showed stable lamina fixation (Figs. 2H, I). MRI demonstrated neural decompression (Fig. 2J), particularly at L2-3 and L5-S1 (Figs. 2K, L). CT confirmed significant spinal canal enlargement and bony regrowth/reunion at the L3 and L4 laminar decompression grooves (Figs. 2M, N).

## Discussion

We demonstrate that bilateral laminoplasty effectively decompresses neural structures with minimal blood loss, fewer transfusion and satisfactory clinical outcomes. This technique bridges the gap between lumbar fusion procedures (which remove compressive elements but cause tissue damage and motion loss) and minimally invasive laminectomy/laminotomy (which may inadequately address multi-level stenosis). Laminectomy with facetectomy/foraminotomy carries instability risks [23], while literature remains inconclusive regarding optimal techniques. Our laminoplasty offers three key advantages: (1) Preserving the facet joints maintains spinal stability by preventing iatrogenic instability, (2) reconstruction of an enlarged osseous spinal canal with potential bony regrowth, and (3) reduced blood loss/hospitalization time with fewer surgery- or comorbidity-related complications. These attributes make it ideal for elderly patients with ≥ 3-level LCS.

Degenerative LCS predominantly affects adults aged ≥ 60 years [5, 6]. Our study focused on symptomatic ≥ 3-level LCS in this demographic, where outcomes are often compromised by comorbidities (hypertension: 53.8%; diabetes: 19.2%) and severe degeneration. Diagnosis required both: (1) CT/MRI-confirmed neural compression, and (2) correlative clinical symptoms. Most cases involved mixed central/lateral recess stenosis, though exact narrowing locations weren’t classified. Given the relative rarity of ≥ 3-level involvement (44.6% of symptomatic LCS [6]), our study provides valuable insights.

Our technique was adapted from cervical microendoscopic laminoplasty [24]. By bilaterally enlarging the spinal canal and fixing the dissociated spinous process to lamina with mini-plates, we achieved decompression while preserving anatomy. Reports of low axial pain, bony healing in decompression grooves, and disc herniation diminishment [25–27] supported this approach for multi-level LCS. Due to the differing anatomical characteristics between the lumbar and cervical spine, decompression grooves were positioned medially rather than at the lateral recess, therefore termed as SALD. We also observed profound disc resorption post-decompression, exceeding natural history expectations [28]. To accommodate greater lumbar biomechanical stresses, we used pre-contoured titanium mini-plates with 4-screw fixation per plate, maximizing stability.

Lumbar stability was maintained without postoperative stiffness as established by multi studies [22, 29–31]. By preserving posterior stabilizing structures (supraspinous/interspinous ligaments) and avoiding facet joint resection, our approach minimized instability risks. Subsequent bony healing further reinforced stability, consistent with animal models showing osseous union at 4–8 weeks [29] and clinical studies reporting minimal instability (5.9%) after reconstruction techniques [22]. Laminectomy alone has negligible impact on sagittal alignment [30], and we observed no instability. Critically, avoiding rigid instrumentation prevented adjacent segment disease (ASD), which affects ≤ 30% of fusion patients [32]. Preserved segmental motion indicated favorable biodynamics. We also measured the stability of lumbar spine by dynamic X-ray detection showing no stability happening in the follow-up.

Various techniques for lumbar spinal canal reconstruction/laminoplasty have been proposed, primarily for tumors, LCS, and lumbar disc herniation. Initially described for LCS/disc herniation in 1991 [33], one technique involved hemilaminectomy with disc resection followed by lamina replantation and screw fixation. Subsequently, inverse laminoplasty [34, 35] was prospectively applied to enlarge the spinal canal in LCS patients by cutting the inner lamina and reattaching it using miniplates; the reattached lamina measured 3.5 mm thick [19]. Another technique involved removing, rotating, and reattaching the spinous process and lamina [36]. Yaser et al. [37] reported a spinous process–splitting laminoplasty designed to minimize muscular trauma, utilizing a minimally invasive approach for undercutting the inner lamina and facet joint. While this technique facilitated exposure via spinous process splitting, we employed an open bilateral laminotomy approach without excessive blood loss. Endoscopic bilateral laminoplasty also represents a feasible minimally invasive option for performing decompression and fixation.

Scholars have adopted bone grafting strategies to reinforce posterior support during reconstruction [18, 38, 39]. For instance, a single open-door laminoplasty technique with posterior iliac bone grafting was used for lumbar stenosis [38], yielding satisfactory results but with interlaminar fusion in 41% of cases and adjacent disc lesions in 11%. Fixation methods like wire [18] or sutures [39] were used, and good clinical outcomes were observed, although fusion was not universally achieved [18]. We elected against bone grafting in our technique due to concerns about adjacent segment degeneration following interlaminar fusion [40] and potential secondary compression from the graft. Evidence indicates that bony healing within decompression grooves can occur without grafting [41–43]. For example, laminoplasty using translaminar screws demonstrated solid bony healing in 2 of 5 patients on CT [41]. Factors like lamina thinning and the space between the spinous process and lamina may limit fusion potential. One study linked bone fusion formation to ODI scores and pain [21]. A method similar to ours using ARCH plates for intraspinal tumors reported bony healing in 70.8% of patients [43], consistent with another study showing healing in 5 of 8 patients (62.5%) [42]. Our representative case also demonstrated solid bony healing across the approximately 3-mm gap within the decompression groove.

This retrospective study has some limitations. This single-center retrospective study had significant loss to follow-up (40.3%). Prospective randomized trials comparing laminoplasty to PLIF, laminectomy, and fusion are needed. Longer follow-up (> 5 years) will assess durability and ASD rates. Minimally invasive endoscopic refinement requires development. Despite limitations, bilateral laminoplasty proves suitable for elderly patients with multi-level LCS, offering excellent outcomes with minimal complications. Its motion-preserving design represents a promising alternative to fusion.

## Supplementary Information

Supplementary Material 1.

## Data Availability

The datasets generated during and/or analyzed during the current study are available from the corresponding author on reasonable request.
