# Supplementary material for: A new surgery choice of bilateral laminoplasty for symptomatic three or more-level lumbar canal stenosis in patients over 60 years old: a two-year retrospective study
Source: BMC Musculoskelet Disord. 2025 Jul 14;26:680. doi: 10.1186/s12891-025-08940-1 (PMC12257856; doi:10.1186/s12891-025-08940-1)
Supplement: Supplementary file 1 — Supplementary Material 1. [file 12891_2025_8940_MOESM1_ESM.docx]

**SI 1 Clinical characteristics of enrolled patients treated by bilateral laminoplasty**

| **Number** | **Gender** | **Age** | **Time of Follow-up** | **symptom** | **Duration of illness** | **Levels of LSS** | **Total blood loss** | **Operation time** | **JOA score** | | **VAS score** | | **ODI score** | | **Comorbid conditions** | **Note** |
| --- | --- | --- | --- | --- | --- | --- | --- | --- | --- | --- | --- | --- | --- | --- | --- | --- |
|  |  |  |  |  |  |  |  |  | **Pre-** | **Post-** | **Pre-** | **Post-** | **Pre-** | **Post-** |  |  |
| 1 | Female | 60 | 21 | Low back pain  Numbness of lower limb | 4 | L3-5 | 100 | 1.5 | 11 | 17 | 7 | 3 | 37% | 31% | Hypertension | 120/70mmHg (Perioperation) |
| 2 | Male | 61 | 21 | Weakness of right limb | 36 | L2-5 | 200 | 2.5 | 21 | 24 | - | - | 35% | 32% | - | - |
| 3 | Female | 62 | 22 | Low back pain  Pain of left lower limb | 4 | L3-5 | 100 | 1 | 23 | 25 | 8 | 4 | 31% | 27% | - | - |
| 4 | Male | 62 | 23 | Low back pain  Numbness of left lower limb | 12 | L2-5 | 200 | 2 | 20 | 17 | 5 | 6 | 33% | 31% | Ankylosing spondylitis | Low back Pain continued |
| 5 | Female | 63 | 22 | Low back pain  Pain of left lower limb | 3 | L3-5 | 100 | 1 | 23 | 25 | 6 | 4 | 23% | 23% | Femoral head necrosis,  Cerebral infarction | Pain alleviated but  continued |
| 6 | Female | 64 | 24 | Sensory dysfunction of left lower limb | 4 | L2-5 | 200 | 2.5 | 16 | 21 | - | - | 26% | 21% | Hypertension,  Osteoporosis | 180/100mmHg (Perioperation) |
| 7 | Female | 65 | 22 | Low back pain  Numbness of right lower limb | 72 | L3-5 | 150 | 1 | 23 | 26 | 7 | 3 | 34% | 22% | Hypertension, Diabetes, Coronary atherosclerosis | 160/100mmHg (Perioperation) |
| 8 | Female | 66 | 24 | Sensory dysfunction of right lower limb | 6 | L3-5 | 100 | 1.5 | 21 | 26 | - | - | 21% | 15% | Hypertension, Hyperuricemia | 130/90mmHg (Perioperation) |
| 9 | Female | 66 | 26 | Low back pain  Numbness of lower limbs | 6 | L2-5 | 200 | 3 | 13 | 16 | 7 | 7 | 35% | 35% | Hypertension, 0steoarthritis, Osteoporosis,  Cerebral infarction,  Coronary atherosclerosis | 160/100mmHg (Perioperation)  Revisited our hospital in 3 months |
| 10 | Male | 68 | 23 | Pain of left lower limb | 12 | L2-5 | 200 | 2 | 23 | 27 | 7 | 2 | 29% | 21% | lymphoma | Chemotherapeutics applied with drugs control cancer pain, Revisited our hospital in 3 months |
| 11 | Male | 69 | 21 | Low back pain  Pain of left lower limb | 3 | L2-5 | 200 | 3 | 21 | 26 | 7 | 2 | 39% | 32% | Hypertension, Diabetes,  Nephropathy | Transferred to ICU due to low SaO_2_ |
| 12 | Female | 70 | 23 | Pain and numbness of left lower limb | 7 | L3-5 | 150 | 2 | 17 | 26 | 6 | 1 | 43% | 31% | Hypertension,  Coronary atherosclerosis | 180/110mmHg (Perioperation) |
| 13 | Female | 71 | 30 | Low back pain  Pain of lower limbs | 5 | L2-5 | 200 | 3 | 21 | 25 | 8 | 3 | 41% | 17% | Coronary atherosclerosis,  0steoarthritis |  |
| 14 | Female | 72 | 31 | Low back pain  Weakness of lower limbs | 60 | L3-5 | 150 | 2 | 15 | 23 | 4 | 3 | 47% | 35% | Coronary atherosclerosis,  Ankylosing spondylitis,  Atrial fibrillation | Low back Pain alleviated but  Continued. |
| 15 | Female | 74 | 24 | Low back pain  Pain of lower limbs | 3 | L2-5 | 200 | 2 | 24 | 26 | 5 | 2 | 41% | 32% | Femoral head necrosis | Pain alleviated but  Continued. |
| 16 | Female | 60 | 23 | Low back pain  Numbness of lower limbs | 24 | L3-5 | 100 | 2 | 15 | 22 | 6 | 0 | 36% | 20% | Diabetes | >10 mmol/L  (Perioperation) |
| L | Female | 75 | 22 | Pain of right lower limb | 4 | L2-5 | 200 | 3 | 26 | 26 | 8 | 3 | 49% | 24% | Parotid adenoma |  |
| 18 | Female | 81 | 29 | Low back pain | 1 | L3-5 | 100 | 1 | 25 | 25 | 8 | 2 | 24% | 19% | Hypertension |  |
| 19 | Female | 85 | 24 | Low back pain  Pain of left lower limb | 1 | L3-5 | 150 | 2 | 21 | 24 | 7 | 5 | 43% | 30% | Diabetes, Hypertension | 170/90mmHg (Perioperation) |
| 20 | Female | 72 | 37 | Low back pain, Sensory dysfunction of right lower limb | 12 | L1-5 | 200 | 3 | 15 | 21 | 6 | 1 | 41% | 22% | Hypertension, anemia | 150/100mmHg (Perioperation) |
| 21 | Male | 66 | 26 | Low back pain  Numbness of lower limbs | 12 | L3-5 | 150 | 3 | 22 | 26 | 6 | 2 | 50% | 32% | Coronary atherosclerosis, Ankylosing spondylitis, |  |
| 22 | Male | 74 | 29 | Pain and numbness of lower limbs | 36 | L1-5 | 250 | 3.5 | 14 | 19 | 5 | 1 | 39% | 24% | Hypertension | 150/90mmHg (Perioperation) |
| 23 | Female | 61 | 27 | Pain and numbness of lower right limb | 3 | L3-5 | 150 | 2 | 22 | 27 | 7 | 2 | 23% | 17% | Hypertension,  Coronary atherosclerosis | 180/100mmHg (Perioperation) |
| 24 | Female | 61 | 27 | Pain and weakness of lower left limb | 12 | L1-5 | 200 | 3 | 21 | 24 | 6 | 2 | 19% | 15% | Hypertension, | 140/100mmHg (Perioperation) |
| 25 | Male | 60 | 24 | Pain and weakness of lower right limb` | 1 | L3-5 | 100 | 2 | 23 | 25 | 4 | 4 | 25% | 21% | Hypertension, Diabetes, | 140/90mmHg (Perioperation) |
| 26 | Male | 70 | 22 | Pain and numbness of lower right limb | 8 | L3-5 | 100 | 2 | 22 | 27 | 5 | 2 | 41% | 32% | Diabetes | >10 mmol/L  (Perioperation) |

*Age (years), Duration of illness (months), Follow-up time (months), Intraoperative total blood loss (ml), operationtime (h), Oxygen saturation=SaO_2_

**SI 2 Clinical characteristics of enrolled patients treated by PLIF**

| **Number** | **Gender** | **Age** | **Time of Follow-up** | **symptom** | **Duration of illness** | **Levels of LSS** | **Total blood loss** | **Operation time** | **JOA score** | | **VAS score** | | **ODI score** | | **Comorbid conditions** | **Note** |
| --- | --- | --- | --- | --- | --- | --- | --- | --- | --- | --- | --- | --- | --- | --- | --- | --- |
|  |  |  |  |  |  |  |  |  | **Pre-** | **Post-** | **Pre-** | **Post-** | **Pre-** | **Post-** |  |  |
| 1 | Female | 60 | 23 | Low back pain  Numbness of left lower limb | 12 | L3-5 | 150 | 3.5 | 27 | 28 | 5 | 0 | 11% | 5% | Hypertension | 130/80mmHg (Perioperation) |
| 2 | Male | 61 | 22 | Low back pain  Numbness of right lower limb | 24 | L3-5 | 200 | 3 | 16 | 20 | 7 | 4 | 17% | 21% | Hypertension | 140/90mmHg (Perioperation) |
| 3 | female | 66 | 23 | Low back pain  Numbness and weakness of lower limbs | 12 | L2-S1 | 800 | 4 | 17 | 20 | 6 | 7 | 32% | 41% | Ankylosing spondylitis | Intraoperative transfusion：  2u of PRBCs  400ml of FFP  Low back Pain  Continued. |
| 4 | female | 66 | 24 | Low back pain  weakness of lower limbs | 5 | L2-5 | 800 | 4 | 16 | 22 | 8 | 4 | 52% | 35% | Osteoarthritis | Intraoperative transfusion：  300ml of FFP |
| 5 | male | 66 | 23 | Pain of left lower limb | 1 | L2-5 | 300 | 3.5 | 22 | 27 | 7 | 3 | 32% | 25% | Hypertension, Diabetes, Osteoarthritis | Intraoperative transfusion：  4u of PRBCs |
| 6 | female | 68 | 25 | Low back pain  Pain of lower limbs | 12 | L3-S1 | 600 | 4 | 18 | 24 | 7 | 7 | 47% | 53% | Hypertension,  Osteoporosis | Intraoperative transfusion：  300ml of FFP  Low back Pain  Continued. |
| 7 | female | 68 | 21 | Low back pain  weakness of right lower limb | 60 | L2-5 | 500 | 4 | 19 | 25 | 8 | 4 | 42% | 30% | Hypertension, Diabetes | Intraoperative transfusion：  400ml of FFP |
| 8 | female | 70 | 22 | Low back pain  Sensory dysfunction of right lower limb | 2 | L2-5 | 400 | 3.5 | 24 | 29 | 5 | 5 | 27% | 20% | Osteoporosis | Intraoperative transfusion：  400ml of FFP |
| 9 | male | 72 | 22 | Low back pain | 1 | L3-5 | 300 | 3 | 21 | 26 | 6 | 3 | 32% | 25% |  |  |
| 10 | female | 73 | 23 | Low back pain  Pain numbness of left lower limb | 3 | L3-5 | 200 | 3.5 | 22 | 26 | 6 | 5 | 34% | 35% | Hypertension, Osteoarthritis | Low back Pain  Continued. |
| 11 | female | 75 | 25 | Pain and numbness of left lower limb | 2 | L3-S1 | 150 | 2 | 20 | 17 | 6 | 6 | 40% | 38% | Hypertension, Diabetes, | Transferred to ICU due to nosocomial pneumonia  Revisited our hospital in 3 months  Pain continued |
| 12 | female | 76 | 24 | Low back pain  weakness of lower limbs | 60 | L2-5 | 50 | 2.5 | 17 | 24 | 5 | 2 | 47% | 30% | Hypertension, Diabetes | Intraoperative transfusion：  2u of PRBCs  400ml of FFP |
| 13 | female | 77 | 36 | Low back pain  Pain of right lower limb | 60 | L2-5 | 500 | 4 | 19 | 25 | 7 | 3.5 | 42% | 28% | Diabetes | Intraoperative transfusion：  100ml of FFP |
| 14 | female | 70 | 21 | Low back pain  Pain of right lower limb | 1 | L3-S1 | 500 | 3.5 | 22 | 23 | 6 | 3 | 32% | 35% | Hypertension, | Intraoperative transfusion：  400ml of FFP |
| 15 | female | 70 | 20 | Low back pain  Pain of right lower limb | 3 | L2-5 | 100 | 3.5 | 23 | 27 | 6 | 1 | 30% | 24% |  |  |
| 16 | male | 70 | 24 | Pain numbness of left lower limb | 2 | L2-5 | 300 | 4 | 20 | 25 | 7 | 3.5 | 37% | 25% | Hypertension, Coronary atherosclerosis, Osteoporosis | Intraoperative transfusion：  100ml of FFP |
| L | female | 71 | 27 | Low back pain  Numbness of left lower limb | 60 | L3-5 | 200 | 3 | 19 | 24 | 7.5 | 4 | 44% | 28% | Diabetes |  |
| 18 | male | 61 | 33 | Pain of lower limbs  Numbness of right lower limb | 12 | L3-5 | 200 | 2.5 | 18 | 23 | 7 | 4 | 42% | 30% | Hypertension |  |
| 19 | male | 77 | 19 | Pain of left lower limb | 2 | L3-5 | 100 | 2 | 23 | 28 | 6 | 3 | 27% | 20% |  |  |
| 20 | female | 78 | 21 | Low back pain  Pain numbness of lower limbs | 60 | L3-5 | 300 | 3 | 16 | 16 | 7 | 4 | 52% | 45% | Coronary atherosclerosis, Diabetes, Osteoporosis | Intraoperative transfusion：  2u of PRBCs  400ml of FFP  Pain continued |
| 21 | male | 81 | 33 | Low back pain | 2 | L3-5 | 300 | 3 | 21 | 26 | 6 | 3 | 37% | 25% |  |  |
| 22 | female | 82 | 25 | Low back pain  Pain of left lower limb | 1 | L2-5 | 400 | 3.5 | 18 | 24 | 8 | 4 | 42% | 58% | Hypertension, Diabetes, Coronary atherosclerosis, | Intraoperative transfusion：  2u of PRBCs  400ml of FFP  Transferred to ICU due to low SaO_2_ |
| 23 | male | 79 | 33 | Low back pain  Pain of left lower limb | 4 | L3-5 | 300 | 3 | 20 | 25 | 7 | 3.5 | 38% | 46% | Hypertension, Diabetes, Coronary atherosclerosis, | Intraoperative transfusion：  2u of PRBCs  400ml of FFP  Transferred to ICU due to low SaO_2_ |
| 24 | female | 60 | 22 | Low back pain  Pain of right lower limb | 2 | L3-5 | 100 | 2 | 16 | 19 | 6 | 1 | 27% | 21% |  |  |
| 25 | female | 62 | 23 | Low back pain  Pain of lower limbs | 12 | L2-5 | 200 | 2.5 | 17 | 18 | 6 | 1 | 36% | 26% | Hypertension |  |

*Age (years), Duration of illness (months), Follow-up time (months), Intraoperative total blood loss (ml), operationtime (h), Oxygen saturation=SaO_2,_ PRBCs=Packed red blood cells, FFP=Fresh frozen plasma
